# Human Oral Phase Coupled with *In Vitro* Dynamic Gastrointestinal Digestion for Assessment of Plant Sterol Bioaccessibility from Wholemeal Rye Bread

**DOI:** 10.1021/acs.jafc.4c02109

**Published:** 2024-07-01

**Authors:** Nerea Faubel, Reyes Barberá, Guadalupe Garcia-Llatas

**Affiliations:** Nutrition and Food Science Area, Faculty of Pharmacy and Food Sciences, University of Valencia, Av. Vicente Andrés Estellés s/n, 46100 Burjassot, Spain

**Keywords:** simgi, phytosterols, solid food, *in vitro* digestion

## Abstract

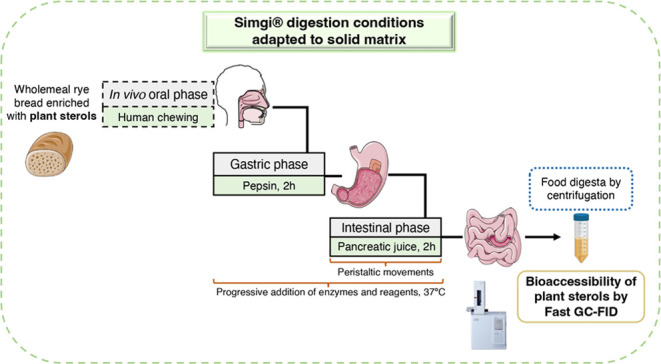

A dynamic gastrointestinal digestion system (simgi) after a human oral phase was used, for the first time, to assess the bioaccessibility of plant sterols (PS) from wholemeal rye bread (74.8 ± 2.2 mg of PS/100 g d.m.) and PS-enriched wholemeal rye bread (PS-WRB) (1.6 ± 0.04 g of PS/100 g of fresh bread). The use of these solid food matrices requires a novel adaptation of the gastric phase of the system. The PS identified in the breads are campesterol, campestanol, stigmasterol, β-sitosterol, sitostanol, Δ5-avenasterol, Δ5,24-stigmastadienol, Δ7-stigmastenol, and Δ7-avenasterol. The bioaccessibility of the total PS, only quantifiable in PS-WRB, is 19.9%, with Δ7-avenasterol being the most bioaccessible and Δ5-avenasterol being the least (*p* < 0.05). As shown in this study, PS-WRB can be considered to be a good choice to include in the daily diet. Furthermore, although the use of dynamic digestion methods for evaluating bioaccessibility implies high costs and technical complexity, their application means a closer approximation to *in vivo* scenarios.

## Introduction

Plant sterols (PS) are one of the bioactive compounds, which promote a better state of health through anti-inflammatory, antioxidant, antidiabetic, and antiproliferative actions,^1^ as well as a reduction in LDL cholesterol levels and the prevention of cardiovascular diseases. It has been shown that an intake of 1.5–3 g of PS could reduce cholesterol in plasma concentration by around 12%.^2^ To reach the intake that provides these effects, the European Union has allowed the enrichment of rye bread with PS as the only solid food matrix.^3,4^

Rye is of great interest due to its nutritional composition, with a high amount of insoluble fiber.^5^ When combined with the effect of PS, it can produce a decrease in cholesterol serum. In this regard, Söderholm et al.^6^ carried out a study in humans confirming cardiovascular protection (intake of 99 or 198 g of PS-enriched rye bread, corresponding to 2 or 4 g of PS daily, respectively).

When optimizing the design of a functional food, in addition to determining the PS content in the food, it is also necessary to know its bioavailability. *In vivo* methods are the best tool to estimate this parameter; however, they have an expensive and ethically restricted process.^7^ In the *in vitro* methods, the first step of digestion is the release of components from the food matrix. For hydrophobic compounds, such as PS, this process includes their solubilization in mixed micelles within the intestinal lumen in a suitable form for absorption through the intestinal wall defined as bioaccessibility.^8,9^ The *in vitro* oro-gastrointestinal digestion methods are used for bioaccessibility evaluation of bioactive compounds, as these methodologies are less expensive and easier to reproduce. The static methods are the most cost-effective and widely used because the digestion conditions are fixed between phases. In order to harmonize the oro-gastrointestinal conditions, a consensus static digestion method has been proposed (INFOGEST 2.0 method).^10^ On the other hand, the dynamic models reproduce gastrointestinal digestion conditions more closely to *in vivo* scenario, providing peristaltic movements and progressive addition of enzymes and reagents, as well as control of the temperature and pH.^11^

There are several types of dynamic digestion methods that have been used over the years, one of which is the simgi, a multicompartmental system with five parts (stomach; small intestine; and ascending, transverse, and descending colon), providing the bioaccessible fraction from the small intestine and also the fermentation liquids from colonic fermentation.^12^

In the case of sterols present in food and food models, only two studies have specifically used the dynamic gastrointestinal digestion model (simgi) on cholesterol bioaccessibility. The influence of soluble fiber from a chia seed mucilage suspension on cholesterol bioaccessibility in a lipid food matrix (100 mg of cholesterol with 1 g of refined olive oil) has been evaluated.^13^ Moreover, a food model mixing wine and lipids (9.9 g olive oil with 343.8 mg of cholesterol added) has been used to assess the interaction between wine polyphenols and cholesterol bioaccessibility.^14^ However, the bioaccessibility of PS has never been assessed using a dynamic digestion method applied to solid food matrices, such as wholemeal rye bread (WRB) (PS-enriched or nonenriched). In plant foods, such as cereals, lipids can remain entrapped within the cells of the plant tissue at later stages of digestion.^15^ The starch content in cereals could disrupt the digestion process; therefore, the use of amylases to hydrolyze glycosidic bond in starch,^9^ as well as to introduce small cracks or fissures during oral processing,^15^ can facilitate the release of bioactive compounds.

In a previous study by our research group,^16^ the bioaccessibility of free PS in PS-enriched wholemeal rye bread has been evaluated. It has been indicating the necessity of a human *vs in vitro* oral phase, providing a better homogenization and accessibility of the digestive enzymes for releasing and incorporating of the PS into the mixed micelles.

In dynamic gastrointestinal models, one of the problems when using solid food matrices could be the difficulty in reproducing the complex gastric emptying of food.^17^ It has been indicated that food structure is not always considered, and solid food should be subjected to a physical dispersion with an ultraturrax blender or mastication simulator before digestion to avoid clogging the tubes of the system.^18^

The evaluation of PS bioaccessibility in cereal-based foods is scarce. Using a static *in vitro* digestion model, the bioaccessibility of steryl ferulates and the content of free sterols in flours and breads (white wheat and mixed with milling fraction flours) have been evaluated.^19^ In oat granola bars containing varying amounts of fat (0, 7, and 24 g/100 g), only bioaccessibility of total phytosterols has been assessed using the INFOGEST method.^20^ A modification of the INFOGEST 2.0 model (considering the use of gastric lipase and cholesterol esterase) has been applied to determine bioaccessibility of individual and total PS in a PS-enriched wholemeal rye bread.^16^

For a better approximation of the *in vivo* situation, the aim of this study is, for the first time, to assess the bioaccessibility of PS in a solid food matrix (WRB and PS-WRB) after a human oral phase and gastrointestinal digestion using a dynamic digestion model (simgi).

## Materials and Methods

### Chemicals

Commercial wholemeal rye flour was sourced from HARINERA LA META S.A. (part of La Meta Group, the Vall Companys Group’s flour division, Barcelona, Spain). The PS ingredient used consisted of microencapsulated free PS (with a purity of 74.7%, w/w) derived from tall oil (Lypophytol ME dispersible, palm-free) and a blank ingredient without PS containing only the excipients used for the microencapsulation were provided by Lipofoods (Barcelona, Spain). l-Ascorbic acid (purity ≥99.0%, w/w) was purchased from Merck LifeScience S.L.U. (St. Louis, MO). The enzymes of digestion used were pepsin from porcine gastric mucosa (ref: P6887) and pancreatin from the porcine pancreas (ref: P1625) purchased from Merck LifeScience S.L.U. (Madrid, Spain), as well as difcoTM Oxgall Dehydrated Fresh Bile (ref: 212820) bought from Thermo Fisher Scientific (Madrid, Spain). The PS standards used were 5β-cholestan-3α-ol (epicoprostanol, 99.8% as internal standard (IS)) (ref: 123663), which was bought from Merck LifeScience S.L.U. (Madrid, Spain), as well as 5,22-cholestadien-24-ethyl-3β-ol (stigmasterol, 97.4%) (ref: S2424) and 24α-ethyl-5α-cholestan-3β-ol (sitostanol, 67.5%) (ref: S462330). 5-Cholesten-24β-ethyl-3β-ol (β-sitosterol, 98.8%) (ref: BP0237) and 24α-methyl-5-cholesten-3β-ol (campesterol, 98.6%) (ref: BP0307) were purchased from Chengdu Biopurify Phytochemicals Ltd. (Sichuan, China). Anhydrous pyridine (10113641) from Acros Organics (Geel, Belgium) and *N*,*O*-bis(trimethylsilyl)-trifluoroacetamide (BSTFA) [1% trimethylchorosilane (TMCS)] T6381 from Merck LifeScience S.L.U. (Madrid, Spain) were purchased as derivatization reagents. Potassium hydroxide (484016) and hydrogen chloride (1003172500) (purity 37%) were purchased from Merck LifeScience S.L.U. (Madrid, Spain). Ethanol (20821296) was provided by VWR (Briare, France). Hexane (HE02342500), diethyl ether (ET00792500), and cyclohexane (CI00392500) were bought from Scharlau (Barcelona, Spain). Water purification was performed with a Milli-Q system (Milford, MA).

### Sample Preparation

The WRB and PS-WRB were made following the methodology by Makran et al.^21^ For the PS-WRB, the dough consisted of 300 g of wholemeal rye flour, 2.5% compressed yeast (based on flour weight), 1.6% sodium salt (based on flour weight), water (adjusted for optimal absorption, 500 BU, 67% based on flour weight), 0.01% ascorbic acid (based on flour weight), and 4.3% of the flour weight accounted for by the PS-containing ingredient. The WRB was prepared by substituting the PS-containing ingredient with a blank ingredient containing only excipients (1.1% based on flour weight). The ingredients were mixed with rotary blades for 11 min, followed by a 10 min resting period. The dough was then divided into four pieces (100.7 or 102.5 g for WRB and PS-WRB, respectively), hand-balled, and allowed to rest for 15 min. The dough fermentation process was done for 45 min at 28 °C with 85% relative humidity, during which it was monitored by periodically measuring the increase in dough volume using graduated cylinders containing 50 g pieces of the remaining dough. Finally, the fermented doughs were baked at 180 °C for 25 min. The chemical composition of the breads is stated elsewhere.

### Oral Phase and Simulated Dynamic Gastrointestinal Digestion

A portion of WRB or PS-WRB (81.45 ± 1.14 g) was chewed, as described by Faubel et al.^16^ Six volunteers (four males and two females, age range: 22–42 years) took part in the *in vivo* study and gave their informed consent to participate. After the screening tests explained by Faubel et al.,^16^ the volunteer who achieved the food/saliva ratio of 1:1 (w/w) or 100% increase of the bolus was defined as the optimal volunteer, with a bolus consistency not thicker than tomato or mustard paste.^10^

For the subsequent digestion phases (gastric and intestinal), the simgi system, developed by the CIAL (CSIC-UAM) (Madrid, Spain), was used, as described by Tamargo et al.,^12^ with some modifications. This system consists of five compartments with different pH maintained at each phase with NaOH and HCl (stomach 1.8, small intestine 7, ascending colon 5.6, transverse colon 6.3, and descending colon 6.8), using a constant temperature (37 °C) and enzyme solution flow. In this study, the model was adapted by carrying out the gastric phase in a double-jacketed glass reactor vessel instead of the original gastric compartment. This adaptation was justified due to the inability to perform peristaltic movements, as well as issues related to gastric emptying, which led to the blockage of the system’s tubes. These facts were attributed to the solid food matrix (WRB), since food structure has been previously identified as a challenge,^18^ in addition to the observation of the high insoluble fiber content (15.3–15.8 g/100 g in fresh bread). Gastric emptying was manually incorporated into the small intestine compartment of the simgi, and magnetic stirring (at 800 rpm in gastric and 1000 rpm in intestinal reactors) was employed to homogenize and attempt to mimic peristaltic movements. The progressive addition of enzymes and solutions to maintain pH was performed as usual in the simgi system.^12^

First, an oral bolus corresponding to 81.45 ± 1.14 g of WRB or PS-WRB was introduced into the stomach. Gastric juice (2000 U/mL pepsin dissolved in a total volume of 15 mL of 150 mM NaCl at a rate of 3.9 mL/min) was added automatically to the stomach compartment to start the gastric phase, which lasts 2 h. The small intestine phase was also performed for 2 h by adding intestinal/pancreatic juice (40 mL at a rate of 5 mL/min), consisting of pancreatin (0.9 g/L) and Oxgall dehydrated fresh bile (6 g/L).

After the intestinal phase, the digesta was centrifuged (Eppendorf centrifuge 5810R, Hamburg, Germany) at 3100 g, 4 °C, and 90 min to obtain the supernatant, which corresponds to the bioaccessible fraction (BF).^16^ The bioaccessibility (defined as the amount of an ingested compound that is potentially available for absorption and is dependent only on digestion and release from the food matrix)^22^ of PS was estimated as a percentage of the PS present in the BF compared to those present in the respective breads (undigested) as follows:
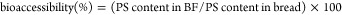


### Determination of PS

**Figure 1:**
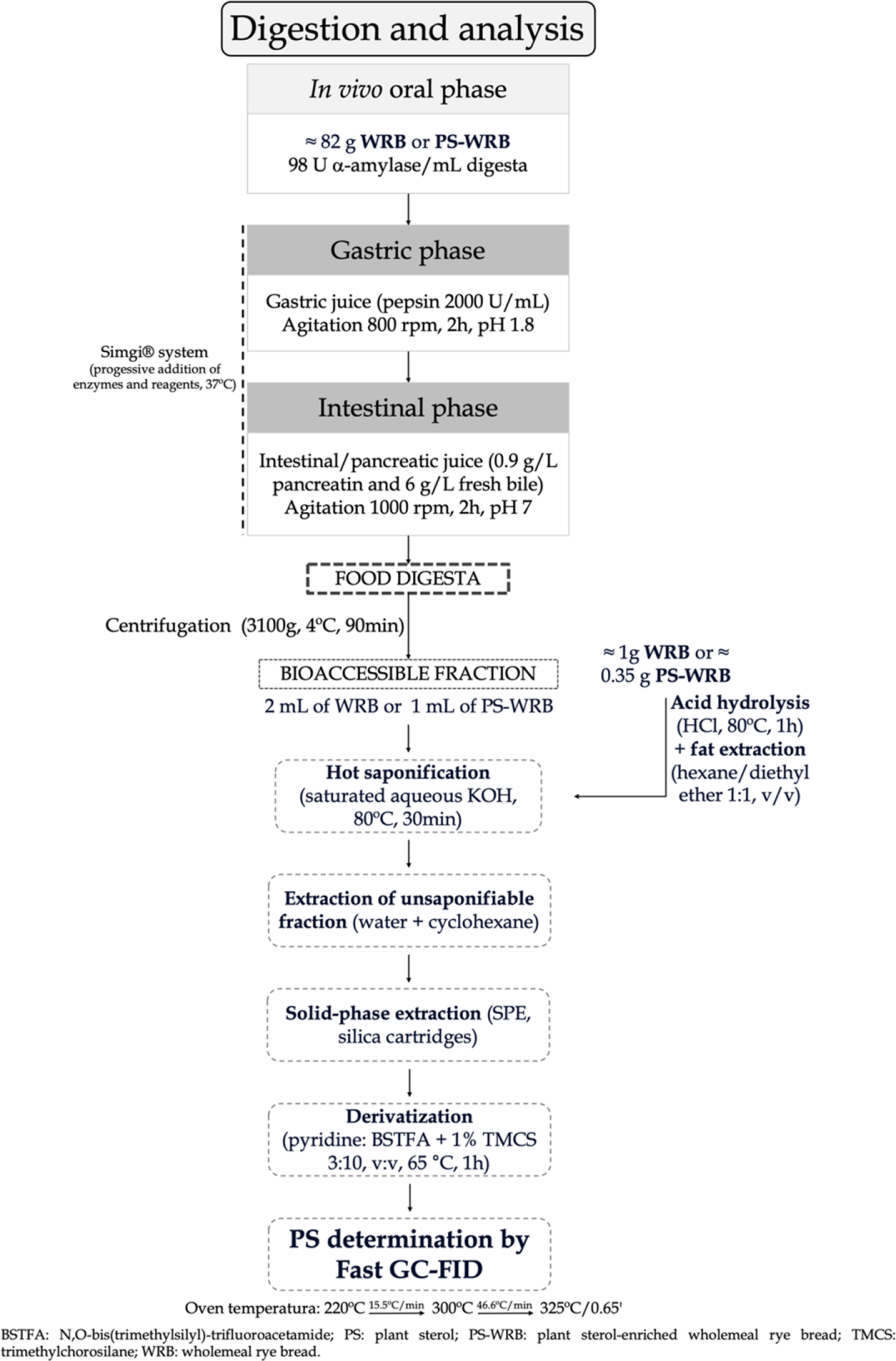
Process flowchart of the bread digestion and analysis of plant sterols.

The methodology used for the determination of PS in WRB, PS-WRB, and their BF was conducted according to Faubel et al.^16^ with slight modifications. The IS (40 or 200 μg) was added to 1 or 0.35 g of partially dried-milled WRB or PS-WRB, respectively. Absolute ethanol was added to the samples, and the samples were subjected to acid hydrolysis and fat extraction. Hot saponification was applied to the fat extracted and 2 mL of BF from WRB (added with 40 μg of IS) or to 1 mL of BF from PS-WRB (added with 200 μg of IS). The extraction of the unsaponifiable fraction was done according to Faubel et al.^16^ and the derivatization step was applied using pyridine/BSTFA + 1% TMCS (3:10, v/v) at 65 °C (SBH200D Blockheater, Stuart, Staffordshire, United Kingdom) for 1 h.^23^ Finally, samples were dissolved in 100 (WRB) or 500 μL (PS-WRB) of hexane and analyzed (0.7 μL) by a FAST gas chromatography-flame ionization detector (Shimadzu GC-2025, Kyoto, Japan) equipped with a Restek Rxi-5Sil MS (10 m × 0.10 mm × 0.10 μm film thickness, Bellafonte, Pennsylvania). An oven was initially programmed at 220 °C, heated to 300 °C at a rate of 15.5 °C/min, and then increased to 325 °C at a rate of 46.6 °C/min, maintaining for 0.65 min. The carrier gas was hydrogen (28.7 mL/min). The split ratio applied was 1:40 and the temperature of the injector and detector was 325 °C. Calibration curves with the PS standards were used for the quantification (Table S1). To quantify Δ5-avenasterol, Δ5,24-stigmastadienol, Δ7-stigmastenol, and Δ7-avenasterol, a β-sitosterol calibration curve with lower points of calibration was developed^24^ since no commercial standards are available. In addition, sitostanol and campestanol were quantified by using sitostanol curves. The whole process flowchart of the bread digestion and analysis of PS is shown in Figure 1.

### Limits of Detection and Quantification for PS Determination in WRB and PS-WRB

Six blanks of 1 or 0.35 g of Milli-Q water, as for WRB and PS-WRB, respectively, were submitted to the same methodology as for PS determination in bread samples. The limits were calculated according to US Food and Drug Administration guidelines:^25^ Limit of detection (LOD) = 3SD/*S* and limit of quantification (LOQ) = 10SD/*S* (where SD is the standard deviation of the method blanks and *S* is the slope of the calibration curve) (Table S2).

### Statistical Analysis

One-way analysis of variance (ANOVA) and Tukey’s post hoc test were employed to assess statistically significant differences (*p* < 0.05) in the bioaccessibility between individual PS for PS-WRB. The entire study was conducted using Graphpad Prism 9.5.1 (GraphPad Software Inc., San Diego, CA).

## Results and Discussion

### Identification and Quantification of PS in WRB and PS-WRB

**Figure 2:**
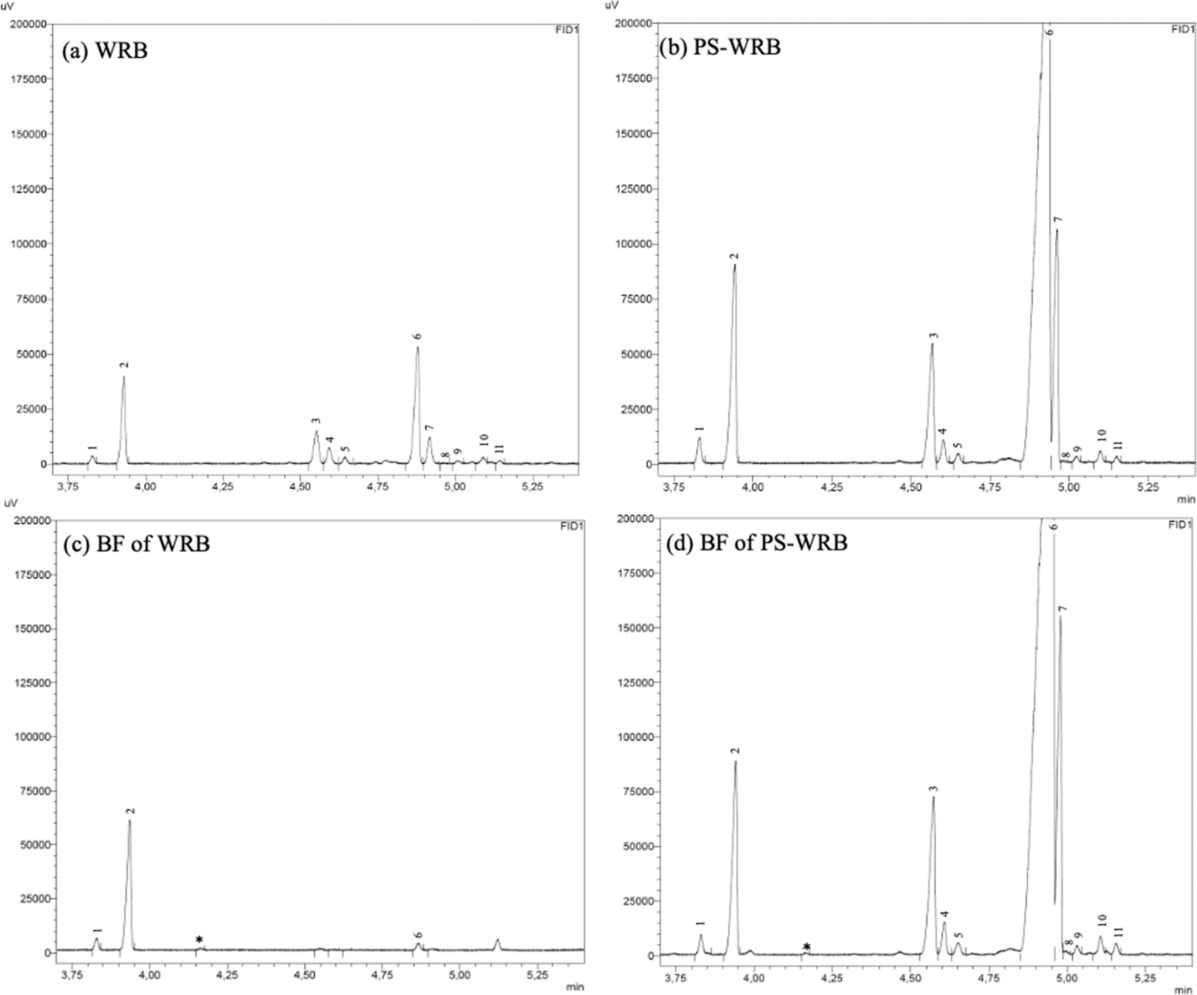
Chromatograms obtained from the wholemeal rye bread (WRB) (a) and plant sterol-enriched wholemeal rye bread (PS-WRB) (b) and their bioaccessible fractions (BFs) (c,d).

The identification of PS in WRB and PS-WRB is shown in Figure 2. It is important to indicate the identification of coprosterol, an impurity present in the IS (epicoprostanol), which is also identified by other authors, who indicate that the quantification of the PS has been done with the sum of both areas of the IS and its impurity.^26,27^

The PS identified in WRB and PS-WRB (Figure 2) are campesterol, campestanol, stigmasterol, β-sitosterol, sitostanol, Δ5-avenasterol, Δ5,24-stigmastadienol, Δ7-stigmastenol, and Δ7-avenasterol. To our knowledge, only two studies identified PS in unfortified rye bread. The same PS as those in our study are identified in rye bread (without specifying if it is wholemeal),^24^ as well as brassicasterol and cycloartenol. However, in a light and dark rye bread, only campesterol, campestanol, stigmasterol, β-sitosterol, and sitostanol were identified.^28^ As in previous studies by our group, Δ5,24-stigmastadienol and Δ7-stigmastenol have also been identified as artifacts of Δ5-avenasterol and β-sitosterol, respectively, due to alkaline hydrolysis and high temperatures applied for PS determination in rye bread.^16^ This fact has been previously described during the processing of wheat and rye bran^29^ and the refining of olive oil.^30^

The same PS identified in bread are present in rye flour and grain, as expected.^24,28^ Other minor sterols (stigmastadienol, gramisterol, α-amyrin, cycloartenol, Δ7-stigmastenol, and citrostadienol), in addition to those indicated by Piironen et al.,^24^ have been identified in wholemeal rye flour.^29,31,32^ The presence of 24-methylcycloartanol and brassicasterol in whole rye grain was detected depending on the studied cultivar. However, in rye flour and bread only brassicasterol is detected.^24^

The total PS content in WRB is 74.8 mg/100 g d.m. (Table 1). When compared with other authors (Table 2), Normén et al.^28^ indicated a lower PS content (51.0 mg/100 g d.m.) in light and dark rye bread in relation to our study and higher contents (136.5 mg/100 g d.m.) in rye bread.^24^ The differences observed do not seem to be attributed to the methodology used for the determination of PS since it is similar. Other possible factors such as different cultivars of rye grain or a different proportion of flour in bread making (not reported in the studies) could be the cause of these variabilities. Regarding the flour (Table 2), the total PS content determined in different wholemeal rye flours^24,29,31^ is between 90.1–142.0 mg/100 g d.m., while in rye flour,^28^ it is lower (86.0 mg/100 g d.m.). Differences in the PS content depending on the part of the rye kernel (endosperm, germ, and bran) have been indicated.^29^ Only the PS value of the bran (176.7 mg/100 g d. m.) has been specified, with this being the one with the highest PS content, which explains how a different proportion of these fractions could imply different amounts of PS in the flour and thus in the rye bread.^29^ Moreover, the total PS content of rye grain has been determined in 10 different cultivars,^24^ reporting a range of 77.4–93.7 mg/100 g d.m. Variations in contents may be due to growing conditions, location, cultivar years, and genetic variations. Finnish variants (Akusti, Riihi, Anna, and Voima) show similar contents (87.9–90.5 mg/100 g d.m.), while the breeding line variants (Bor 7068, 9214, and 9414) show higher variability (84.6–93.7 mg/100 g d.m.). The lowest PS contents (77.4 and 78.0 mg/100 g d.m.) are observed in the German hybrid cultivars (Esprit and Picasso, respectively). Similar values of total PS (69.0 and 99.5 mg/100 g d.m.) have been reported in rye grains,^28,29^ while lower content (26.2 mg/100 g d.m.) has been indicated when applying different PS determination methodology (direct extraction of the fat fraction without acid hydrolysis).^33^

**Table 1:** Content of Plant Sterols in the Wholemeal Rye Bread (WRB)^a^

plant sterol	WRB (mg/100 g d.m.)
campesterol	11.6 ± 0.5 (15.5)
campestanol	11.7 ± 0.4 (15.7)
stigmasterol	1.9 ± 0.1 (2.5)
β-sitosterol	28.5 ± 0.9 (38.1)
sitostanol	17.3 ± 0.3 (23.2)
Δ5-avenasterol	0.3 ± 0.02 (0.4)
Δ5,24-stigmastadienol	0.9 ± 0.02 (1.2)
Δ7-stigmastenol	1.8 ± 0.1 (2.4)
Δ7-avenasterol	0.8 ± 0.1 (1.0)
total PS	74.8 ± 2.2

a Data expressed as mean ± standard deviation (*n* = 6). Relative percentage of all sterols between parentheses.

**Table 2:** Average Content (mg/100 g d.m.) of Plant Sterols in Rye Breads, Flours, and Grains Found in the Literature

	breads			flours					grains			
plant sterol	rye^24^	rye (light)^28^	rye (dark)^28^	wholemeal rye (sampling 1/sampling 2)^24^	rye^28^	special dark^29^	wholemeal rye,^31^^a^	wholemeal rye,^32^^a^	rye^28^	whole rye,^24^^a^	whole rye^29^	rye^33^
brassicasterol	2.5									-/tr		
campesterol	24.2	11.0	11.0	20.9/16.2	17.0	23.1	18.8–25.9	18.0–22.2	14.0	14.0–16.3	16.7	5.4
campestanol	11.3	3.6	3.7	8.6/6.7	7.3				0.0	6.3–8.0		0.9
stigmasterol	4.4	2.2	2.2	3.7/3.2	3.3		3.0–5.5	3.4–4.1	2.4	2.4–2.8		1.4
β-sitosterol	69.3	29.0	28.0	54.7/46.3	48.0	67.1	50.8–71.2	51.2–62.1	42.0	39.2–36.8	48.5	14.5
sitostanol	15.0	5.1	5.0	11.9/8.6	11.0				11.0	7.0–11.3		1.9
Δ5-avenasterol	4.2			2.1/1.6						1.0–1.8		
cycloartenol + Δ7-stigmastenol	4.8			3.8/-								
Δ7-avenasterol	2.3			1.7/1.4						1.0–1.5		
24-methylcycloartanol				-/0.6						-/tr-0.8		
stanols				–/–		26.3	16.3–24.9	18.6–21.1			17.9	
others				-/5.7^b^		21.1	12.1–18.1	14.8–17.1		4.6–6.4	16.5	1.84^c^
total PS	136.5	51.0	51.0	106.0, 90.1	86.0	137.5	109.8–142.0	108.3–123.3	69.0	77.4–93.7	99.5	26.2

a Different cultivars.

b Not reporting the name of other sterols.

c Δ7-sitosterol and unidentified sterol.

The abundance of individual PS (Table 1) in WRB is as follows: β-sitosterol > sitostanol > campestanol = campesterol > stigmasterol > Δ7-stigmastenol > Δ5,24-stigmastadienol > Δ7-avenasterol > Δ5-avenasterol. When compared with other studies,^24,28^ campesterol values are more abundant than stanols in our work. The β-sitosterol and campesterol contents in the WRB are similar in relation to Normén et al.,^28^ while the campestanol and sitostanol contents are similar to those reported by Piironen et al.^24^ The content of Δ7-avenasterol in WRB is higher (0.8 mg/100 g d.m.) than that of Δ5-avenasterol (0.3 mg/100 g d.m.) (Table 1), whereas an opposite abundance has been reported^24^ (2.3 and 4.2 mg/100 g d.m., respectively) (Table 2). In the present study, and for the first time, the content of individual β-sitosterol artifacts (Δ5,24-stigmastadienol and Δ7-stigmastenol) in rye breads has been determined. Only Piironen et al.^24^ indicate the quantification of Δ7-stigmastenol + cycloartenol, reporting a higher content (4.8 mg/100 g d.m.) versus our study (1.8 mg/100 g d.m.). Regarding the PS-WRB, a total PS content of 1.6 g/100 g of fresh bread is detected (Table 3). Only two studies by our research group^16,34^ have determined the PS content in other PS-WRB samples, with it being 1.4-fold higher than in the PS-WRB of our study.

**Table 3:** Content of Plant Sterols in the Plant Sterol-Wholemeal Rye Bread and Their Bioaccessible Fraction and Bioaccessibility^a^

	PS-WRB		
plant sterol	bread	BF	bioaccessibility
	mg/100 g fresh bread		%
campesterol	122.53 ± 4.49 (7.66)	25.00 ± 0.57 (7.91)	20.40 ± 0.47a
campestanol	33.84 ± 1.61 (2.12)	6.65 ± 0.25 (2.11)	19.66 ± 0.74a
stigmasterol	8.43 ± 0.29 (0.53)	1.68 ± 0.11 (0.53)	19.97 ± 1.31a
β-sitosterol	1143.84 ± 29.24 (71.53)	226.57 ± 5.80 (71.73)	19.81 ± 0.51a
sitostanol	272.71 ± 6.63 (17.05)	52.22 ± 1.68 (16.53)	19.15 ± 0.62 a
Δ5-avenasterol	0.92 ± 0.04 (0.06)	0.13 ± 0.03 (0.04)	14.50 ± 3.23b
Δ5,24-stigmastadienol	4.34 ± 0.33 (0.27)	0.77 ± 0.05 (0.24)	17.64 ± 1.08ab
Δ7-stigmastenol	8.54 ± 0.75 (0.53)	1.78 ± 0.11 (0.56)	20.86 ± 1.30a
Δ7-avenasterol	3.98 ± 0.23 (0.25)	1.06 ± 0.07 (0.34)	26.72 ± 1.64c
total PS	1599.13 ± 42.92	315.87 ± 8.50	19.86 ± 1.15

a Relative percentage of all sterols between parentheses. BF: bioaccessible fraction; PS: plant sterols; PS-WRB: plant sterol-enriched wholemeal rye bread. Plant sterol content in the bread (*n* = 6) and BF (*n* = 3), and bioaccessibility (sterol content in bioaccessible fraction × 100/sterol content in bread) are expressed as mean ± standard deviation. Different lowercase letters indicate statistically significant differences (*p* < 0.05) between bioaccessibility of PS (a-c).

The order of abundance of individual PS in PS-WRB is β-sitosterol > sitostanol > campesterol > campestanol > Δ7-stigmastenol = stigmasterol > Δ5,24-stigmastadienol > Δ7-avenasterol > Δ5-avenasterol, similar to that indicated for WRB (Table 1) and previous studies.^16,34^ The contents of campesterol, β-sitosterol, Δ7-stigmastenol, and Δ7-avenasterol (Table 3) in PS-WRB are lower (1.3–2.6-fold) than those indicated by Faubel et al.^16^ and Miedes et al.^34^ and even lower for Δ5-avenasterol (17- and 23-fold, respectively). Stigmasterol and Δ5,24-stigmastadienol show no differences, whereas a higher campestanol (1.4- and 1.9-fold, respectively) and sitostanol (1.2-fold) levels are observed in PS-WRB.^16,34^

### Bioaccessibility of WRB and PS-WRB

Figure 2 shows the chromatograms of the BF from the breads. As a novelty, a dynamic simulated gastrointestinal digestion of WRB and PS-WRB has been carried out in order to obtain the bioaccessibility of PS. The PS contents in the BF of WRB are below the LOD and LOQ (Table S2) (except for β-sitosterol, which can be identified but not quantified), so it is not possible to calculate the bioaccessibility. Moreover, cholesterol is also detected, which is provided by the digestion reagents (pancreatin extract and bile) reported in a previous study.^35^

Only one study on wheat flour and bread^19^ has determined the content and bioaccessibility of steryl ferulates and the content of free sterols. Whole grain wheat flour or flour with 70% wheat baking flour and 30% wheat milling fraction with a high content of steryl ferulates and free sterols has been assayed. A static simulated digestion method was applied with the addition of pepsin enzyme, pancreatin, and bile as well as lipase (not included in our study), reporting that the bioaccessibility of steryl ferulates is less than 0.1%. The authors indicated that the endogenous lipase that may have been present in the flour was activated during digestion, promoting the reduction of steryl ferulates in whole wheat and mixed flour (74 and 75%, respectively). Although it is possible that during the baking process, the lipase was denatured, with a lower reduction of steryl ferulates being observed in both breads (14 and 16%, respectively).^19^ The total free sterol content increase was 187–214% after digestion of both flours and breads. This increase in total free sterols may be due to the hydrolysis of steryl ferulates by a pancreatin extract containing lipase and cholesterol esterase. The same trend was observed by these authors in a previous study,^36^ in which the same digestion was performed on different grains (polished, cargo, and wild rice, as well as rice, corn, and wheat bran), indicating a decrease in steryl ferulates, and in turn, an increase in free sterols.

The same PS identified in the PS-WRB (Table 3) are quantified in the corresponding BF. Only one other previous study^16^ has determined the PS in the BF obtained after a static digestion method (INFOGEST) from another PS-WRB sample. The main difference compared to the present study is the equipment used for digestion, as our study uses a dynamic model, simgi, with the progressive addition of enzymes and solutions to control pH. Moreover, there are also differences in the pH of the gastric phase (3 static vs 1.8 dynamic) and in the concentration of enzymes added to the intestinal phase (pancreatin 100 U/mL vs. 0.9 g/L and bile salts 10 mM vs bile 6 g/L, respectively). A 1.6-fold lower content of total PS in the BF of PS-WRB (315.87 mg/100 g fresh bread) has been observed versus the static method,^16^ decreasing the PS bioaccessibility 1.2-fold (19.86%). These lower contents and different bioaccessibility values could be attributed to the lower initial PS content in our PS-WRB and different digestion methodologies (dynamic vs static).

The relative abundance of individual PS in the BF of the PS-WRB (Table 3) is β-sitosterol > sitostanol > campesterol > campestanol > Δ7-stigmastenol = stigmasterol > Δ7-avenasterol > Δ5,24-stigmastadienol > Δ5-avenasterol. This order of abundance is similar to that observed for the PS-WRB, except for Δ7-avenasterol being higher than Δ5,24-stigmastadienol. The highest bioaccessibility was obtained for Δ7-avenasterol (26.72%), while the lowest is for Δ5-avenasterol and Δ5,24-stigmastadienol (14.50 and 17.64%). The bioaccessibility of the rest of the PS (19.15–20.86%) shows no statistically significant differences between them (*p* < 0.05) (Table 3), although the content of individual PS in rye bread before digestion is variable.

The bioaccessibility of PS in another cereal-based food (granola bars enriched with 1.5 g PS/100 g) has been evaluated considering different forms of PS enrichment (PS encapsulated in nanoporous starch aerogel (NSA), PS + empty NSA, and PS + pregel starch) and amounts of fat (0, 7, and 24 g/100 g granola bars).^20^ After a static digestion method, the bioaccessibility obtained for the nonfat (0 g/100 g) and low fat (7 g/100 g) ranged from 16 to 88%, which includes the bioaccessibility values obtained for our sample containing 3.4 g fat/100 g PS-WRB. However, it can be considered that the food matrix evaluated (PS-enriched granola bars vs PS-WRB), the digestion methodology (static vs dynamic simulated digestion), and the addition of enzymes (fungal lipase and pepsin vs only pepsin in gastric phase) are different in these studies.

In conclusion, the applied methodology proves to be advantageous, as it better replicates physiological conditions, with a human oral phase (more physiological than mechanical methods) and a dynamic gastrointestinal digestion system (simgi). However, two limitations must be considered, namely, the need to adapt the gastric phase to solid matrices, such as bread, as well as the costs and sophisticated technology of this equipment. The WRB enriched with PS provides an alternative to the conventional consumption of wheat bread as part of a regular diet since its insoluble fiber content together with the PS enrichment could help to decrease cardiovascular risk.

## Supporting Information Available

Table S1: calibration curves with plant sterol standards obtained by FAST GC-FID; Table S2: limits of detection and quantification for wholemeal rye bread and PS-wholemeal rye bread (PDF)

## Abbreviations

BF: bioaccessible fraction
BSTFA: *N*,*O*-bis(trimethylsilyl)-trifluoroacetamide
FAST GC-FID: FAST gas chromatography-flame ionization detector
IS: internal standard
LOD: limit of detection
LOQ: limit of quantification
PS: plant sterols
PS-WRB: plant sterol-enriched wholemeal rye bread
TMCS: trimethylchlorosilane
WRB: wholemeal rye bread
