# Supplementary material for: Human Oral Phase Coupled with In Vitro Dynamic Gastrointestinal Digestion for Assessment of Plant Sterol Bioaccessibility from Wholemeal Rye Bread
Source: J Agric Food Chem. 2024 Jul 1;72(28):15672–9. doi: 10.1021/acs.jafc.4c02109 (PMC11261621; doi:10.1021/acs.jafc.4c02109)
Supplement: Supplementary file 1 — jf4c02109_si_001.pdf [file jf4c02109_si_001.pdf]

Supporting Information

Human oral phase coupled with *in vitro* dynamic gastrointestinal digestion for assessment of plant sterol bioaccessibility from wholemeal rye bread

Nerea Faubel, Reyes Barberá, and Guadalupe Garcia-Llatas*

Nutrition and Food Science Area, Faculty of Pharmacy and Food Sciences, University of Valencia

Av. Vicente Andrés Estellés s/n, 46100 Burjassot, Spain

*Corresponding author: Nutrition and Food Science Area, Faculty of Pharmacy and Food Sciences, University of Valencia Av. Vicente Andrés Estellés s/n, 46100 Burjassot, Spain. E-mail address: Guadalupe.garcia@uv.es

Table S1. Calibration Curves with Plant Sterol Standards Obtained by FAST GC-FID

Table S2. Limits of Detection and Quantification for Wholemeal Rye Bread and PS-Wholemeal Rye Bread

Table S1. Calibration Curves with Plant Sterol Standards Obtained by FAST GC-FID

Plant sterol	Range (µg in assay)	*r*	Calibration curve	Confidence interval of slope (95%)
Campesterol	[a] 8.58-25.64	0.9949	y = 0.0181x + 0.0626	0.0147-0.0215
	[b] 53.24-321-44	0.9990	y = 0.0049x – 0.0594	0.0046-0.0052
Stigmasterol	[a] 2.53-5.06	0.9958	y = 0.0236x – 0.0054	0.0206-0.0266
	[b] 3.80-18.70	0.9983	y = 0.0045x – 0.0031	0.0041-0.0050
β-sitosterol	[a] 42.28-79.04	0.9905	y = 0.0302x – 0.1334	0.0244-0.0360
	[b] 1067.04-2766.40	0.9996	y = 0.0073x – 2.0724	0.0069-0.0076
	[a] 0.30-5.95*	0.9985	y = 0.025x – 0.0018	0.0225-0.0275
	[b] 0.30-29.80*	0.9998	y = 0.005x + 0.0008	0.0049-0.0052
Sitostanol	[a] 7.97-31.73	0.9973	y = 0.0094x – 0.006	0.0081-0.0107
	[b] 230.26-474.20	0.9999	y = 0.0041x – 0.3085	0.0040-0.0042
	[c] 12.79-127.87	0.9975	y = 0.0026x + 0.0006	0.0021-0.0032

a: Calibration curves carried out with 40 µg of epicoprostanol, used for quantification of WRB and its BF. b: Calibration curves carried out with 200 µg of epicoprostanol, used for quantification of PS-WRB and its BF. c: Calibration curves carried out with 200 µg of epicoprostanol and used for quantification of campestanol in PS-WRB and its BF. BF: bioaccessible fraction; PS-WRB: plant sterol-enriched wholemeal rye bread; WRB: wholemeal rye bread. β-

Sitosterol curves indicated with and asterisk (*) were developed for the quantification of Δ5-avenasterol, Δ5,24-stigmastadienol, Δ7-stigmastenol and Δ7-avenasterol.

Table S2. Limits of Detection and Quantification for Wholemeal Rye Bread and PS-Wholemeal Rye Bread

Plant sterol	WRB				PS-WRB			
	LOD		LOQ		LOD		LOQ	
	μg (in assay)	mg/100 g	μg (in assay)	mg/100 g	μg (in assay)	mg/100 g	μg (in assay)	mg/100 g
Campesterol	0.906	0.453	3.021	1.511	0.679	0.969	2.262	3.232
Campestanol	0.395	0.197	1.315	0.658	0.333	0.476	1.111	1.587
Stigmasterol	0.102	0.051	0.339	0.169	0.285	0.407	0.950	1.357
β-Sitosterol	6.543	3.272	21.811	10.906	5.101	7.287	17.003	24.290
Sitostanol	2.749	1.374	9.163	4.581	2.651	3.788	8.838	12.626
Δ5-Avenasterol	0.076	0.038	0.253	0.126	0.268	0.383	0.894	1.277
Δ5,24-Stigmastadienol	0.073	0.037	0.244	0.122	0.158	0.226	0.527	0.753
Δ7-Stigmastenol	0.059	0.030	0.198	0.099	0.298	0.426	0.994	1.420
Δ7-Avenasterol	0.100	0.050	0.335	0.167	0.204	0.291	0.680	0.971

LOD: limit of detection; LOQ: limit of quantification. PS-WRB: plant sterol-enriched wholemeal rye bread; WRB: wholemeal rye bread. LOD and LOQ calculated from blanks (n=6).
